# Urolithin A improves mitochondrial health, reduces cartilage degeneration, and alleviates pain in osteoarthritis

**DOI:** 10.1111/acel.13662

**Published:** 2022-07-01

**Authors:** Davide D'Amico, Merissa Olmer, Andréane M. Fouassier, Pamela Valdés, Pénélope A. Andreux, Chris Rinsch, Martin Lotz

**Affiliations:** ^1^ Amazentis SA, EPFL Innovation Park Lausanne Switzerland; ^2^ Department of Molecular Medicine Scripps Research La Jolla CA USA

**Keywords:** chondrocytes, mitochondria, mitophagy, Mitopure, osteoarthritis, urolithin

## Abstract

Osteoarthritis (OA) is the most common age‐related joint disorder with no effective therapy. According to the World Health Organization, OA affects over 500 million people and is characterized by degradation of cartilage and other joint tissues, severe pain, and impaired mobility. Mitochondrial dysfunction contributes to OA pathology. However, interventions to rescue mitochondrial defects in human OA are not available. Urolithin A (Mitopure) is a natural postbiotic compound that promotes mitophagy and mitochondrial function and beneficially impacts muscle health in preclinical models of aging and in elderly and middle‐aged humans. Here, we showed that Urolithin A improved mitophagy and mitochondrial respiration in primary chondrocytes from joints of both healthy donors and OA patients. Furthermore, Urolithin A reduced disease progression in a mouse model of OA, decreasing cartilage degeneration, synovial inflammation, and pain. These improvements were associated with increased mitophagy and mitochondrial content, in joints of OA mice. These findings indicate that UA promotes joint mitochondrial health, alleviates OA pathology, and supports Urolithin A's potential to improve mobility with beneficial effects on structural damage in joints.

## Graphical abstract

Mitochondrial dysfunction is a hallmark of joint aging and osteoarthritis (OA). Here, we show that the natural compound Urolithin A (UA) improves mitophagy and mitochondrial function in human cartilage cells from healthy donors and OA patients. In an in vivo model of OA, UA improves joint mitochondrial health, reduces inflammation, and protects against OA‐associated cartilage degeneration and pain, indicating the potential of UA to promote joint function and mobility during aging and in OA patients.

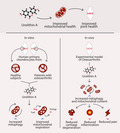

## 1 INTRODUCTION

Osteoarthritis (OA) is the most common joint disease and a leading cause of disability in the western world (Hunter & Bierma‐Zeinstra, 2019). Aging represents a main risk factor for onset and progression of OA (Driban et al., 2020; Hunter & Bierma‐Zeinstra, 2019; Loeser et al., 2016). With the growing aged human population, OA prevalence is increasing and the disease is expected to become one of the most prevalent in the next decade (Turkiewicz et al., 2014). Despite this, there are currently no OA disease‐modifying therapies and pharmacological therapy is limited to pain management (Loeser et al., 2016).

OA progression is characterized by progressive joint tissue destruction and is initiated by disruption of the superficial zone integrity and increased chondrocyte‐mediated extracellular matrix degradation in the cartilage tissue (Hunter & Bierma‐Zeinstra, 2019). Chondrocytes are the only cell type present in mature cartilage and are responsible for function, integrity, and homeostasis of the articular tissue. Chondrocytes have a very low rate of proliferation, making them prone to accumulate damage (Goldring & Goldring, 2007). This causes the progressive decline in their proliferative and synthetic capacity, thereby increasing the risk of OA with aging (Barbero et al., 2004; Loeser, 2009).

Cartilage cells function physiologically in an anaerobic or hypoxic environment where downregulation of mitochondrial respiration by hypoxia‐Inducible Factor 1α is essential for cell survival (Yao et al., 2019). While normal cartilage metabolism is primarily anerobic, mitochondrial dysfunction is a common feature of joints affected by OA (Blanco & Rego‐Pérez, 2020). Chondrocytes have a lower mitochondrial respiratory capacity in OA compared with healthy cartilage (Maneiro et al., 2003; Wang et al., 2015). One mechanism contributing to reduced mitochondrial metabolism is the impaired ability of joint cells to generate new mitochondria through mitochondrial biogenesis (Wang et al., 2015). In addition, chondrocytes from diseased joints accumulate mitochondria that are dysfunctional, as a consequence of the impaired ability of cells to remove damaged mitochondria, through a process called mitophagy (Palikaras et al., 2018). Mitophagy is impaired in several age‐related diseases and interventions to rescue this deficit showed promising results in conditions such as muscle aging, cardiac and neurodegenerative disorders (Palikaras et al., 2017). Targeting mitophagy is therefore considered a relevant strategy to be explored for the treatment of OA (Sun et al., 2021). However, while interventions have been tested to restore mitochondrial biogenesis (Masuda et al., 2018) and to promote mitochondrial respiration (Ohashi et al., 2021), no pharmacological approach has been investigated so far to rescue mitophagy in preclinical models of OA.

Here, we aim to rescue mitophagy and improve joint health through administration of Urolithin A (UA). UA is a metabolite naturally produced by the gut microbiome from natural polyphenols, ellagic acid (EA), and ellagitannins, following the consumption of several food products including pomegranate, berries, and nuts (D'Amico et al., 2021; Espín et al., 2013).

UA was shown to promote mitophagy and mitochondrial health in the muscle of preclinical models of aging (Ryu et al., 2016) and muscle dystrophy (Luan et al., 2021), leading to improved muscle strength and endurance. UA's health benefits were reported also in other diseases (D'Amico et al., 2021), including inflammatory bowel disease (IBD) (Singh et al., 2019), heart failure (Savi et al., 2017), and neurodegenerative disorders (Fang et al., 2019). Clinical studies showed that UA is safe, bioavailable, improves biomarkers associated with better mitochondrial function (Andreux et al., 2019; Liu et al., 2022; Singh et al., 2022) and significantly increases muscle strength (Singh et al., 2022) and resistance to fatigue (Liu et al., 2022) in humans.

In this study, we investigated UA's impact on mitophagy and mitochondrial function in human knee joint cells from healthy donors and OA patients. Furthermore, we assessed UA's ability to reduce cartilage degradation, blunt inflammation, and alleviate pain in a mouse model of OA. Finally, we looked at the effects on UA on mitochondrial health measuring biomarkers of mitophagy and mitochondrial content in OA joint tissues.

## 2 RESULTS

### 2.1 UA increased mitochondrial respiration and mitophagy in healthy human chondrocytes

**FIGURE 1:**
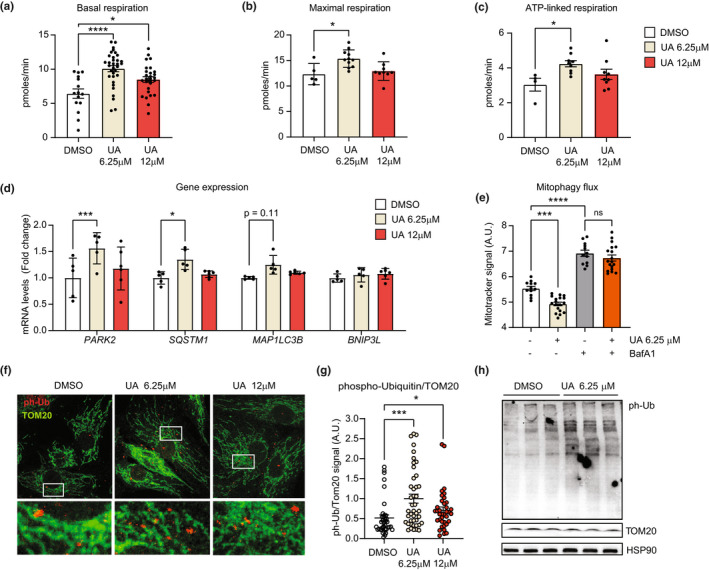
(a–c) Oxygen consumption rates (OCR) showing basal (a) maximal (b) and ATP‐linked (c) mitochondrial respiration in primary healthy chondrocytes (HC), treated with DMSO or Urolithin A (UA) at 6.25 μM and 12 μM for 24 h. (technical replicates for a, 15–33; for b, 5–9; for c, 5–9). *p < 0.05; ****p < 0.0001 after one‐way ANOVA. Error bars represent mean ± SEM. (d) mRNA levels of mitophagy/autophagy genes *PARK2*, *SQSTM1*, *MAP1LC3B*, and *BNIP3* in HC treated with DMSO or the indicated doses of UA for 24 h (*N* = 5). *p < 0.05; ***p < 0.001 after one‐way ANOVA. Error bars represent mean ± SEM. (e) Mitotracker green fluorescent signal, representing the mitochondria quantity in cells treated with DMSO, 6.25 μM UA, 100 nM bafilomycin A1 (BafA1) or cotreated with both 6.25 μM UA and 100 nM BafA1 for 24 h. (*N* = 12–18). ***p < 0.001; ****p < 0.0001 after one‐way ANOVA. Error bars represent mean ± SEM. (f, g) Representative images of HC treated as in (d) for 24 h and stained for TOM20 (green) and phospho‐ubiquitin (ph‐Ub, red). White squares indicate regions corresponding to insets. Nuclei were stained in blue with DAPI (f). Corresponding quantification of the intensity of ph‐Ub over TOM20 (g). (*N* = 27–43) *p < 0.05; ***p < 0.001 after one‐way ANOVA. Error bars represent mean ± SEM. (h) Western blot of HC treated DMSO or UA at 6.25 μM for 24 h and probed with antibodies against phospho‐ubiquitin (ph‐Ub), TOM20 and HSP90 mitochondrial and cellular loading controls

To study the impact of UA on mitochondrial health in joints, we collected primary human chondrocytes (HC) from the knee of a healthy donor and analyzed them using a battery of mitochondrial function and mitophagy assays. First, we measured oxygen consumption rate (OCR) to assess mitochondrial respiration in cells treated with UA or vehicle for 24 h. Two doses of UA were selected (UA 6.25 μM and 12 μM), as they did not show confounding effects on HC cell viability (Fu et al., 2019). UA significantly increased basal (Figure 1a) respiration at both doses and FCCP‐induced, maximal respiration (Figure 1b) and ATP‐linked respiration (Figure 1c) at the lower dose. Proton leak was unaltered (Figure S1a) and also extracellular acidification rate (ECAR), which measures glycolic activity, remained unchanged (Figure S1b), indicating that UA enhances chondrocyte metabolic function by specifically activating mitochondrial respiration.

UA treatment for 24 h did not increase the mRNA expression of either mitochondrial biogenesis or oxidative phosphorylation (OXPHOS) genes (Figure S1c), suggesting that higher mitochondrial function did not derive from increased mitochondria content.

Conversely, UA significantly induced the autophagy/mitophagy markers *PARK2* and *SQSTM1*, genes encoding for Parkin and p62 proteins, at the lower dose (Figure 1d). It also mildly increased levels of *MAP1LC3B*, a gene encoding for the autophagy protein LC‐3. No changes were observed in mRNA levels of *BNIP3* (Figure 1c), a protein promoting mitophagy independently on PINK1‐Parkin. These data suggest that UA enhances mitophagy by activating the PINK1‐Parkin mediated pathway. To confirm this hypothesis, we first checked UA's impact on mitophagy flux, quantifying mitochondrial abundance in cells treated with either UA or UA in combination with bafilomycin A1 (BafA1), an inhibitor of autophagy and mitophagy flux. Treatment with UA alone reduced mitochondrial content, as measured by Mitotracker Green, but co‐treatment with BafA1 abolished this effect (Figure 1e), indicating that UA enhances mitophagy flux in healthy chondrocytes. We then looked at UA‐mediated changes in phospho‐ubiquitin (ph‐Ub) levels. Ph‐Ub molecules are bound to mitochondrial proteins following ubiquitination by Parkin and consequent phosphorylation by PINK1 (Palikaras et al., 2018). An increase in ph‐Ub levels is therefore a specific marker of PINK1‐Parkin mediated mitophagy activation. Immunostaining of HC treated with UA at both tested doses showed a significant increase in ph‐Ub signal, compared to controls (Figure 1f,g). Western blot analysis further validated the increase in ph‐Ub levels in UA compared to vehicle‐treated cells (Figure 1h).

### 2.2 UA increased mitochondrial health in OA‐patient‐derived chondrocytes

**FIGURE 2:**
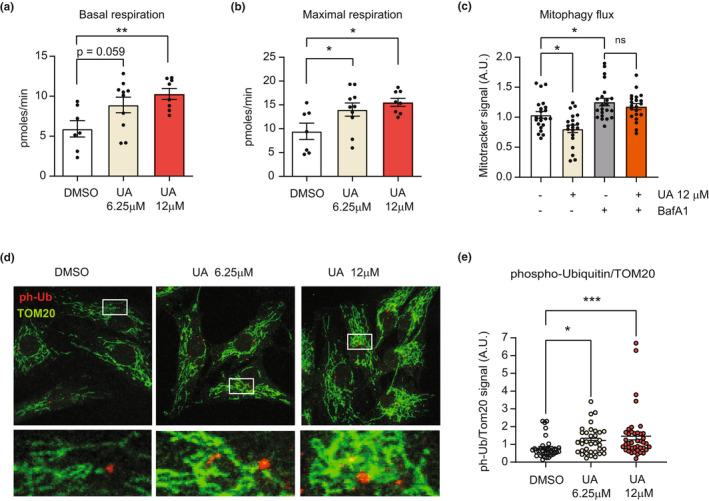
(a, b) Oxygen consumption rates (OCR) showing basal (a) and FCCP‐induced maximal respiration (b) in human primary chondrocytes (HC) from an OA patient, treated with DMSO or UA at 6.25 μM and 12 μM for 24 h (a). (*N* = 7–10). *p < 0.05; **p < 0.01; after one‐way ANOVA. Error bars represent mean ± SEM. (c) Mitotracker green fluorescent signal, in cells treated with DMSO, 12 μM UA,  100 nM bafilomycin A1 (BafA1) or cotreated with both 12 μM UA and 100 nM BafA1 for 24 h. (*N* = 22–24). *p < 0.05, after one‐way ANOVA. Error bars represent mean ± SEM. (d, e) Representative images of HC treated as above for 24 h and stained for TOM20 (green) and phospho‐ubiquitin (ph‐Ub, red). White squares indicate the regions corresponding to insets. Nuclei were stained in blue with DAPI (D). Corresponding quantification of the intensity of ph‐Ub over TOM20 (E). (*N* = 35–40) *p < 0.05; ***p < 0.001 after one‐way ANOVA. Error bars represent mean ± SEM

We further tested whether UA had beneficial effects on mitochondria also in a primary HC cell line derived from a patient with OA. Seahorse analysis was performed in same conditions as for healthy chondrocytes. Mitochondrial respiration was lower in diseased compared to healthy HC (Figure 1a versus Figure 2a), consistently with previous reports (Maneiro et al., 2003; Wang et al., 2015). Notably, UA treatment led to a significant increase in both basal (Figure 2a), maximal (Figure 2b), and ATP‐linked (Figure S2a) mitochondrial respiration in OA chondrocytes, in a dose‐dependent manner. These data suggest that higher doses of UA are more effective to rescue mitochondrial defects in diseased compared with healthy joints. Proton leak (Figure S2b) and basal glycolytic activity of OA HC was unaltered by UA treatments (Figure S2c). As observed for healthy HC, better respiration in OA HC was also accompanied by enhanced mitophagy flux (Figure 2c) and increased levels of mitophagy, assessed by ph‐UB staining (Figure 2d,e).

These data indicate UA's ability to increase PINK1‐Parkin mediated mitophagy and mitochondrial respiration in chondrocytes from both healthy individuals and subjects affected by OA.

### 2.3 UA protected against cartilage degradation in vivo in a surgically induced model of OA

**FIGURE 3:**
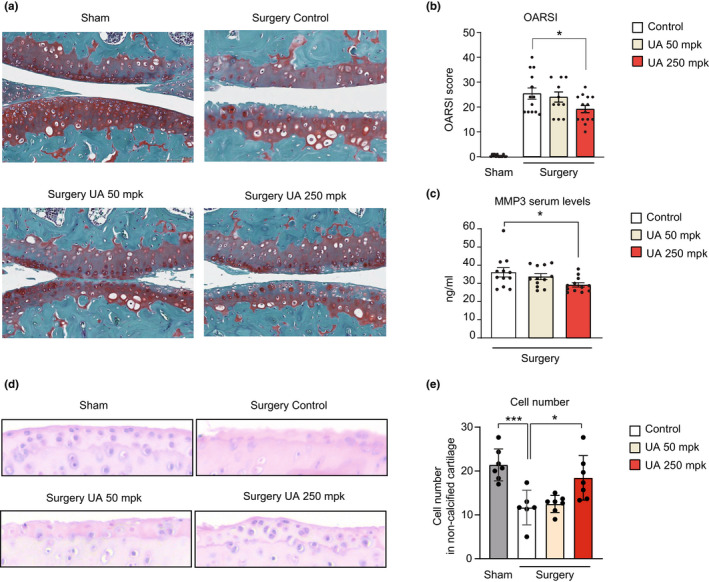
(a) Representative images of safranin‐O staining of knee joints of sham and operated leg. DMM surgery causes cartilage erosion in the middle zone in femur and tibia and additional loss of superficial zone and uncalcified cartilage. Scale bar 100 μM. (b) Mean OARSI score for each mouse, ranging from 0 (no damage) to 48 (maximal degeneration) (*n* = 13 control, *n* = 12 UA 50 mpk, and *n* = 14 UA 250 mpk). *p < 0.05, one‐way ANOVA. Error bars represent mean ± SEM. (c) Serum levels of metalloproteinase 3 (MMP3) in the indicated groups expressed as ng/mL (*n* = 13). *p < 0.05, one‐way ANOVA. Error bars represent mean ± SEM. (d) Representative joint sections form the indicated groups stained with hematoxylin‐eosin (H&E). Magnification: 10 × . (e) Quantitative analysis of cell number from (d) (*N* = 6). *p < 0.05, ***p < 0.001 one‐way ANOVA. Error bars represent mean ± SEM

Cell‐based results prompted us to investigate the effects of UA in vivo in an experimental model of OA. We employed the medial meniscal destabilization (DMM) model that has been broadly used to test therapeutic strategies in rodents and that recapitulates pathological features of OA patients (Little & Hunter, 2013; Malfait & Little, 2015). WT adult mice underwent DMM surgery in the right knee and sham surgery in the left knee. The day after surgery, mice were fed for 8 weeks with a control diet or with diets supplemented with either UA at 50 or 250 mpk (biological replicates: *n* = 13, control diet; *n* = 12, UA 50 mpk and *n* = 14, UA 250 mpk). Knee sections were collected at the end of the study and stained with safranin‐O, a marker of glycosaminoglycan depletion in OA. Following DMM surgery, knee joints exhibited cartilage erosion in the femur and tibia, loss of superficial zone, and decrease of the uncalcified cartilage (Figure 3a). Qualitative analysis of knee joint sections indicated that UA treatment decreased OA pathological changes (Figure 3a). To quantify these changes, sections were blinded and scored following the OARSI semiquantitative grading, that is based on lesion severity and percentage of the area affected in the entire knee, considering both femur and tibia (Pritzker et al., 2006). As expected, the OARSI score increased robustly in knees that underwent surgery, compared to sham (Figure 3b). The analysis of DMM mice treated with UA revealed a dose‐dependent impact on the OARSI score, with significant reduction at the UA 250 mpk dose, compared with the control group (Figure 3b). We further measured serum levels of the metalloproteinase 3 (MMP3), a well‐established blood biomarker associated with increased cartilage degradation (Attur et al., 2013). In line with the histopathological analysis, UA treatment at the 250 mpk dose significantly reduced circulating levels or MMP3 protein after 8 weeks of treatment (Figure 3c). These results demonstrate that administration of UA at 250 mpk for 8 weeks alleviates cartilage degradation in knee joints with OA.

Joint sections were further stained with eosin/hematoxylin (H/E) to quantify cell density (biological replicates = 6 per group). Cell number strikingly decreased in surgery versus healthy knees (Figure 3d,e). However, UA treatment at the higher dose significantly increased cellularity in OA knee joints (Figure 3d,e). Overall, these histopathological analyses illustrate UA's protective role on OA, by reducing cartilage damage and promoting joint cell survival.

### 2.4 UA treatment blunted OA‐related pain and reduced inflammation

**FIGURE 4:**
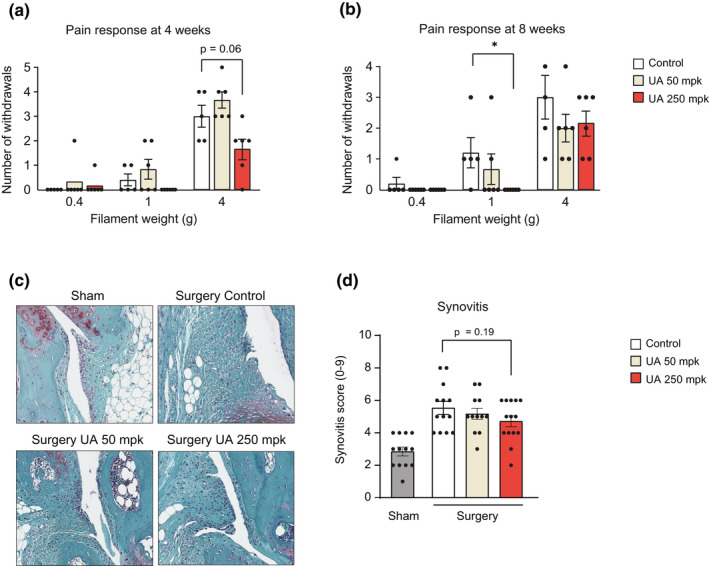
(a, b) Von Frey test performed at 4‐ (a) and 8‐week (B) post‐surgery. The mean withdraw response ± SEM is shown for both legs in each mouse and ranges from 0 (no pain response) to 5 (maximal pain response). Von Frey filaments used correspond to 0.4, 1, and 4 g (*n* = 5 control, *n* = 6 UA 50 mpk, and *n* = 6 UA 250 mpk). *p < 0.05. One‐way ANOVA. Error bars represent mean ± SEM. (c) Representative images of knee synovial membrane in sham and operated leg in the indicated groups, stained with safranin‐O. (d) Quantification of Krenn score to determine synovitis from images as in (c). Data for each mouse range from 0 (no synovitis) to 9 (maximal inflammation) (*n* = 13 control, *n* = 12 UA 50 mpk, and *n* = 14 UA 250 mpk). One‐way ANOVA. Error bars represent mean ± SEM

Severe joint pain is the major clinical OA symptom. To investigate the effect of UA on OA‐related pain, pain‐evoked response was determined 4 and 8 weeks after treatments using the Von Frey test, a nociception assay widely used in OA mice models (Piel et al., 2014, 2015). This test scores the positive paw withdraw response to a painful stimulus using filaments of increasing stiffness. Already 4 weeks after the treatment, UA administration at 250 mpk led to a trend toward the decrease in pain response compared control diet, using the filament of higher stiffness (Figure 4a). Von Frey test assessed at the end of the 8‐week study showed that nociception was significantly blunted by UA at 250 mpk with a thinner filament (Figure 4b) (biological replicates: *n* = 5 control, *n* = 6 UA 50 mpk, and *n* = 6 UA 250 mpk). These data suggest UA can reduce OA‐associated pain and that higher doses or longer treatments could enhance UA's pain relief activity.

Chronic inflammation promotes the progression of human OA and contributes to increased pain (Liu‐Bryan & Terkeltaub, 2015). Therefore, we studied whether improved joint health and reduced pain after UA treatment were associated with decreased inflammation. The inflammation of the synovial membrane, or synovitis, is an active component of OA pathology (Koprich et al., 2017). Synovitis severity was assessed using the Krenn score, which considers three parameters: enlargement of the synovial lining cell layer, density of stroma resident cells and inflammatory infiltrate (Krenn et al., 2002). DMM knees scored higher than sham knees and UA treatment led to a signal toward the decrease in synovitis, at the 250 mpk dose compared to untreated mice (Figure 4c,d) (biological replicates: *n* = 13 control, *n* = 12 UA 50 mpk, and *n* = 14 UA 250 mpk).

### 2.5 UA increased mitophagy in an OA in vivo model

**FIGURE 5:**
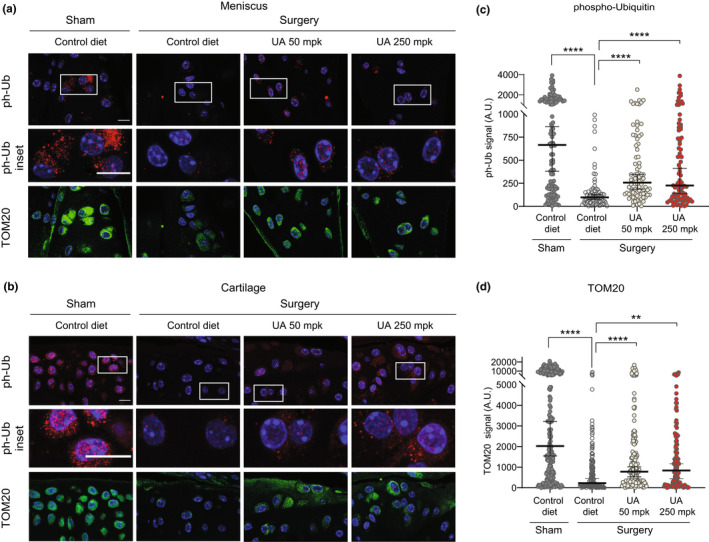
(a) Representative confocal images of meniscus area from the indicated groups stained against phospho‐ubiquitin (ph‐Ub, red) and TOM20 (green). White squares indicate the regions corresponding to insets. Nuclei were stained in blue with DAPI. Scale bar 10 μM. (b) Representative confocal images of cartilage area from the indicated groups (*n* = 6 ) stained as in (a). Scale bar 10 μM. (c, d) Corresponding quantification of ph‐Ub (c) and TOM20 (d) fluorescent signal per cell (arbitrary units) in 2 to 3 independent regions from both meniscus and cartilage area from 4 to 5 mice per group. Each dot represents one cell (*N* = 70–112). **p < 0.01; ****p ≤ 0.0001. One‐way ANOVA. Error bars represent mean ± SEM

Supported by data in cells from OA patients and by previous work in other age‐related conditions (Fang et al., 2019; Ryu et al., 2016), we investigated whether UA was able to improve mitochondrial health in OA joints in vivo. Joint sections were co‐stained for ph‐Ub, to measure mitophagy levels, and TOM20, to quantify mitochondrial content, in both meniscus (Figure 5a and Figure S3a) and cartilage (Figure 5b and Figure S3b). The quantification of both areas showed ph‐Ub signal to strikingly decrease in mice that underwent DMM surgery compared with controls. In diseased animals, treatment with UA at both 50 mpk and 250 mpk doses significantly enhanced absolute intensity of the ph‐Ub mitophagy marker, compared to untreated mice (Figure 5c and Figure S3c) (biological replicates: *N* = 4, control diet; *N* = 5 surgery groups. Number of cells/mouse: *N* = 70–112). TOM20 staining showed similar results, with reduced TOM20 protein level in OA versus healthy knees and a robust increase following UA treatments (Figure 5d). Notably, UA increased joint ph‐Ub levels when normalizing its signal over TOM20, indicating that UA treatments can increase both total mitochondrial content and relative number of mitochondria undergoing mitophagy (Figure S2d).

## 3 DISCUSSION

Mitochondrial dysfunction is a hallmark of OA. However, there are still no mitochondria‐targeting compounds able to treat this disease (Mao et al., 2020). Here, we combine cell‐based and in vivo models to show that the natural, gut‐derived metabolite UA positively impacts joint mitochondrial health, ameliorates OA disease progression, and improves OA‐associated pain.

Past literature supports the potential benefit of mitophagy activators to treat OA. The induction of general autophagy was shown to decrease the severity of experimental osteoarthritis (Caramés et al., 2012) and to protect chondrocytes from oxidative stress (D'Adamo et al., 2020). Genetic studies silencing the mitophagy regulator Parkin in chondrocytes proved that mitophagy is required to maintain mitochondrial quality in OA (Ansari et al., 2018). Finally, a previous in vitro report showed UA to induce mitophagy and protect mouse chondrocytes from mechanical overload injury (He et al., 2021).

Our present work further investigated the translational potential of UA on joint health and disease by employing primary human cells derived from both healthy donors and OA patients. We showed that UA increases mitophagy flux and activates PINK1‐Parkin mitophagy pathway in both cell types. It also provides evidence that mitophagy activation is associated with better mitochondrial respiration, a key feature to support cellular function. Future studies including inhibition of mitophagy components are warranted to help discern UA's specific mechanisms of action in chondrocytes.

Notably, we report improved mitochondrial health by UA for the first time in an in vivo model of OA, with a 2‐month treatment increasing markers of both mitophagy and mitochondrial content in knee joints. This is in line with the coordinated activation of mitophagy and mitochondrial biogenesis observed in muscle cells and tissues after long‐term treatment with UA in preclinical models of aging (Ryu et al., 2016) and muscle dystrophy (Luan et al., 2021) and with the activation of biomarkers linked to improved mitochondrial efficiency in the skeletal muscle in humans (Andreux et al., 2019; Liu et al., 2022, Singh et al., 2022). Muscles and joints are key tissues to support our mobility. Therefore, the combined effect of UA on both joint and muscle mitochondrial health strengthen the translational relevance of this compound to ameliorate OA‐associated disability and support overall mobility.

Several health benefits of Urolithin A have been described for other tissues, such as brain, heart, and intestine as well as for the immune system (D'Amico et al., 2021). This study cannot rule out a broader systemic impact of UA that might also contribute to chondroprotection.

We also showed an effect of UA to reduce pain. Improved pain outcome might contribute to UA's beneficial impact on mobility and warrants follow‐up studies with UA in other debilitating conditions characterized by high pain response, such as cervical spine pain, musculoskeletal injuries, and exercise overload. These UA benefits on pain can be related to the reduced structural damage in the joint tissues or may be a consequence of reduced joint inflammation, a major driver of OA pain (Geraghty et al., 2021). However, our present results do not allow us to draw direct conclusions about mitochondrial changes specifically in sensory neurons associated with pain. Mechanisms responsible for effects of UA on pain remain to be determined.

UA exhibited a mild anti‐inflammatory activity in the experimental setting used in this study. A more robust effect of UA on markers of inflammation was previously reported in the same mouse model (Fu et al., 2019). The differences could derive from distinct experimental conditions used in the studies. The previous work employed gavage administration of UA at 20 mpk, while in our study, UA was given as food admix at 50 mpk and 250 mpk. It is plausible that administration route, dosing, and corresponding PK profiles could influence UA's impact on inflammation. Future studies are warranted to understand the PK/PD activity of UA in the context of OA and the exact contribution of UA's anti‐inflammatory activity or other mechanisms of action, such as effects on Map kinases, Nrf2, or cellular senescence in improving OA disease progression. Additional investigations are needed to examine whether UA only inhibits mechanisms of inflammation and tissue destruction or whether it also has regenerative effects. In the present study, UA's mechanism and effect appear to be chondroprotective rather than chondroreparative. Indeed, cartilage growth does not appear to account for the differences in histopathology, as mice were 4 months old, an age when they have reached maximal size and are considered skeletally mature.

From a translational point of view, thus far there are no drugs that have been successfully tested in clinical trials for simultaneous symptom and disease modification in OA (Katz et al., 2021). This suggests that new therapeutic targets and agents are needed. UA is the only compound tested in preclinical models of OA with the potential to both reduce inflammation and improve mitochondrial health. This evidence, and the lack of effective drugs to target OA, calls for future studies to investigate the impact of UA on OA in clinical settings.

In summary, this work shows the positive impact of UA on mitochondrial health in joints and supports the potential of UA to improve joint function and mobility in both healthy subjects and in patients suffering from OA.

## 4 EXPERIMENTAL PROCEDURES

### 4.1 Cell cultures

Human chondrocytes from healthy donors were purchased from Cell applications (ref. 402‐05a). Human OA chondrocytes were isolated from cartilage that was removed during knee arthroplasty in patients with OA. Fibrillated and macroscopically normal appearing cartilage was included. Cartilage was digested with 0.2% type 2 collagenase overnight. All primary cells were maintained in T75 flasks in ready to use chondrocyte growth medium (Cell application, 411–500), trypsinized and plated directly for the experiments. Trypsin–EDTA 0.05% (VWR, L0930‐500) was used to detach the cells from the flasks. All cells were maintained at 37°C and 5% CO2.

### 4.2 Quantitative real‐time PCR (q‐RT‐QPCR)

RNA from cells was extracted using TRIzol (Thermo Scientific, 15,596,026) and then transcribed to cDNA by the QuantiTect Reverse Transcription Kit (Qiagen, 205,313) following the manufacturer's instructions. The RT‐qPCR reactions were performed using TaqMan master mix (Thermo Fisher 4,369,510). TaqMan probes are listed in Table S1). The analysis was performed with Quantstudio 6 Flex (Life Technologies). All quantitative polymerase chain reaction (PCR) results were presented relative to the mean of housekeeping genes (ΔΔCt method). mRNA levels were normalized over *GAPDH* for gene expression for cell and tissue samples (technical replicates: *N* = 5 from 1 biological replicate).

### 4.3 Seahorse mitochondrial respiration assay

Human chondrocytes from healthy and OA donors were seeded in 96‐well seahorse plates (Agilent Seahorse XF96/XFe96 FluxPak, 102,416–100) and treated the day after with the indicated doses of UA in chondrocyte growth medium (Cell applications, 411–500). After 24 h, the growth medium was replaced by a fatty acid oxidation (FAO) medium (111 mM NaCl, 4.7 mM KCl, 1.25 mM CaCl_2_, 2 mM MgSO_4_, 1.2 mM NaH_2_PO_4_, 10 mM glucose, 1 mM pyruvate, 2 mM glutamine and 0,5 mM L‐Carnitine, pH = 7.4) for 20 min. Oligomycin and Carbonyl cyanide‐p‐trifluoromethoxyphenylhydrazone (FCCP) (Agilent Seahorse XF Cell Mito Stress Test, 103,015–100) were injected in the cartridges (Agilent Seahorse XF96/XFe96 FluxPak, 102,416–100) at a respective final concentration of 1 μM and 2 μM. ATP‐linked respiration was determined by subtracting basal respiration values to the respiration values after oligomycin injection. Antimycin A (AA) and rotenone (Rot) (Agilent Seahorse XF Cell Mito Stress Test, 103,015–100) were injected in the cartridges at 0.5 μM. Proton leak was calculated subtracting respiration values following AA/Rot injection to basal respiration. Technical replicates for experiments in healthy subject‐derived cells: Basal respiration, 15–33; Maximal and ATP‐linked respiration, 5–9. Technical replicates for experiments in OA patient‐derived cells: 7–10.

### 4.4 Mitophagy flux assay

Human chondrocytes from healthy and OA donors were treated with DMSO, UA 6.25 μM or 12 μM, with 100 nM bafilomycin A1 (BafA1) or cotreated with both UA and BafA1 for 24 h. At the end of all treatments, cells were incubated with in serum‐free DMEM with Mitotracker Green (Invitrogen, M7514) at a final concentration of 200 nm for 30 mins at 37°C. Mitotracker signal was acquired after washing the cells with PBS using a plate reader [(Ex (nm) 490/Em (nm) 516, FLUOstar OPTIMA, BMG labtech)]. Immediately after collecting the Mitotracker data, 100uL of Cell titer glo reagent was added to the same plates and a cell viability test was performed following the manufacturer's instruction (CellTiter‐glo Luminescent Cell Viability Assay, Promega, G7571). Background signal from wells without cells was subtracted from all cell‐containing wells. Data represent Mitotracker Green signal normalized over cell number indicated by CellTiter‐glo signal. Technical replicates for experiments in healthy subject‐derived cells, *N* = 12–18 and in OA patient‐derived cells, *N* = 22–24.

### 4.5 Western blot analysis

Cells were lysed in RIPA buffer [50 mM tris (pH 7.4), 1% triton, 0.5% NA‐DCA, 0.1% SDS, 150 mM NaCl, and 2 mM EDTA] with Halt™ protease (Thermo Fisher Scientific, A32955) and phosphatase (Thermo Fisher Scientific, 78,428) inhibitor cocktails (*N* = 3). Proteins were isolated, and the concentration was assessed by DC protein assay (Bio‐Rad, 500–0116). Lysates were eluted in 5× Laemmli buffer (Biorad, 1,610,747) supplemented with 0,1 M DL‐Dithiothreitol (Sigma, D0632‐1 g). Equal amounts of proteins were separated by SDS‐PAGE and transferred onto polyvinylidene difluoride membranes (Biorad, 1,704,156). Filters were washed in TBS + 0.05% Tween (Brunschwig, SER42598‐01) and blocked for 1 h with 5% non‐fat milk (Applichem, A0830‐1 kg). Primary antibodies used are the following: Anti‐phospho‐ubiquitin (Ser65) (Millipore, ABS1513‐I, 1:1000); Tom20 (Santa Cruz, sc‐17,764, 1:1000); and HSP90 (Proteintech, 60,318‐1‐Ig, 1:3000).

### 4.6 Tissue and cell immunofluorescence analysis

Human chondrocytes were fixed with 4% PFA for 20 min, permeabilized with 0.1% Triton‐X‐100, and blocked with 3% BSA 1 h at RT. Cells were stained with phospho‐ubiquitin (Millipore, ABS1513‐I, 1:200) and Tom20 (Santa Cruz, sc‐17,764, 1:150) overnight at 4°C, incubated with secondary antibodies (goat anti‐rabbit Alexa 555, 1:200; goat anti‐mouse Alexa Fluor‐488, 1:200) for 1 h at RT and counterstaining with Dapi (Thermo Scientific, 33,342, 1:10000) for 10 min.

Tissue immunostaining was performed on fresh frozen section of mice knees tissues. 4 μm sections were cut from paraffin‐embedded samples. Sections were de‐waxed and rehydrated, permeabilized in PBS + 0,2% Triton‐X‐100 for 15 min at 37°C and blocked with 5% Goat serum (Jackson Immuno Research), in 1X PBST for 1 h at RT. Sections were stained using antibodies against phospho‐ubiquitin (Millipore, ABS1513‐I, 1:100) and Tom20 (Santa Cruz, sc‐17,764, 1:150) overnight at 4°C and incubated with secondary antibodies (goat anti‐rabbit Alexa 555, 1:200; goat anti‐mouse Alexa Fluor‐488, 1:200) 1 h at RT. A final counterstaining with Dapi (Thermo Scientific, 33,342, 1:10000) was performed for 10 min. Meniscus and cartilage areas were thresholded and fluorescent intensity was assessed per each cell for both phospho‐ubiquitin and Tom20, from random fields. Average cellular signal of phospho‐ubiquitin was also calculated per mouse (*N* = 4, control diet; *N* = 5, UA treated‐mice) and data analyzed after log10 transformation. Image processing was performed with Image J. Knee joint sections (biological replicates: *N* = 6) were stained with hematoxylin and eosin and cellularity assessed as in (Caramés et al., 2012).

### 4.7 Mice

All animal experiments were approved by the Institutional Animal Care and Use Committee at The Scripps Research Institute (TSRI). Pathogen‐free C57BL/6J WT male mice were obtained from the Scripps Division of Animal Resources. Mice were housed in a temperature‐controlled environment with 12‐h light/dark cycles and received food and water ad libitum. Experimental osteoarthritis was induced in 4‐month‐old male C57Bl/6J mice by transection of the medial meniscotibial ligament and the medial collateral ligament in the right knee (DMM mice). The left knee was not subjected to surgery and was used as a control (sham). Mice were terminated after 8 weeks of surgery. Diets containing UA at the doses of 50 mg/kg and 250 mg/kg were provided for 8 weeks post‐surgery. Mice were euthanized after the respective treatments and knee joints collected for histological analysis.

### 4.8 Von Frey testing

The same designated room was used for all behavioral studies, and all tests were performed between 11 am and 3 pm. Mice were placed on a perforated metal floor within small Plexiglas cubicles and were allowed for 15 min of habituation before each test. (*N* = 5 control, *N* = 6 UA 50 mpk, and *N* = 6 UA 250 mpk). The plantar surface of each hindpaw of DMM and of sham paw was stimulated with a series of von Frey hairs (Touch Test fibers #0.4, #1, #4 grams) with log‐arithmetical increments in stiffness and applied perpendicularly to the plantar surface (Tran et al., 2017). Each mouse was tested five times, and the average of the threshold was measured. Observers were blinded to treatment group assignments.

### 4.9 Histological analysis of knee joints cartilage degeneration (OARSI score)

Knee joints were collected from mice with experimental OA (*N* = 13 control, *N* = 12 UA 50 mpk, and *N* = 14 UA 250 mpk). A detailed protocol for tissue collection has been previously described (Caramés et al., 2012 ). Briefly, knee joints were fixed with zinc buffered formalin and decalcified in a Shandon TBD‐2 decalcifier for 48 h. Knee joints were washed with paraffin processed and embedded. Knee joint serial sections (4 μm thick) were stained with Hematoxylin and Safranin O‐fast green for histological analysis using a semiquantitative grading system for cartilage (Pritzker et al., 2006). Briefly, a grading assessment (from 0 to 6) and a stage assessment (from 0 to 4) were used to evaluate tibia and femur. The total OARSI knee score corresponding to the sum of tibia plus femur scores was determined for both knees in each mouse (total knee scale from 0 to 48).

### 4.10 Histological analysis of synovial inflammation and scoring

Synovium from mice with experimental OA was examined using a grading system for synovial inflammation (Krenn et al., 2002) (*N* = 13 control, *N* = 12 UA 50 mpk, and *N* = 14 UA 250 mpk). The scoring system considers three parameters: enlargement of the synovial lining cell layer (ranging from 0 = one layer of cells to 3 = more than 5 layers), density of stroma resident cells (ranging from 0 = normal cellularity to 3 = cellularity greatly increased), and inflammatory infiltrate (ranging from 0 = no inflammatory infiltrate to 3 = very dense inflammatory infiltrate). The score from each parameter was summed for each mouse to provide the total synovitis score (total score from 0 = no synovitis to 9 = maximal synovitis).

### 4.11 Detection of MMP‐3 in mouse serum

At the end of the 8 weeks of treatment, mice underwent a cardiac puncture for terminal blood collection. Samples were collected in polystyrene tubes and allowed to clot during 15 min at RT. Samples were centrifuged at 240 *g* at 4°C for 10 min, and the resulting supernatant was rapidly transferred to clean tubes and immediately stored at −80°C. For MMP‐3 detection, serum samples were analyzed with the commercially available Quantikine total MMP‐3 Elisa kit (R&D Systems) following manufacturer recommendations (*N* = 13). All serum samples were diluted 1:10 in the kit diluent for the analysis and compared to the provided standards.

### 4.12 Statistical analysis

Statistically significant differences between 2 independent groups were determined with Student's unpaired *t* test. Statistically significant differences between three independent groups were determined by one‐way ANOVA test followed by Tukey's multiple comparison test.

## AUTHOR CONTRIBUTIONS

M.L., D.D., P.V., P.A.A., and C.R. contributed to the design of the study. M.O. performed the in vivo study. MO analyzed OARSI scores and Von Frey assay. A.M.F., D.D., P.A.A., and P.V. performed and analyzed cell‐based and immunofluorescence experiments and interpreted all data. D.D. and M.L. wrote the manuscript, with the help of the other co‐authors. All authors reviewed the manuscript.

## CONFLICT OF INTEREST

The authors declare the following competing interests: D.D., A.M.F, and C.R. are current employees and P.V. and P.A.A. were prior employees of Amazentis SA; C.R. is board member of Amazentis SA.

## Supporting information


Table S1

Figure S1

Figure S2

Figure S3

## Data Availability

Data sharing is not applicable to this article as no new data were created or analyzed in this study.
