# Supplementary figures and images for: Urolithin A improves mitochondrial health, reduces cartilage degeneration, and alleviates pain in osteoarthritis
Source: Aging Cell. 2022 Jul 1;21(8):e13662. doi: 10.1111/acel.13662 (PMC9381911; doi:10.1111/acel.13662)

**A**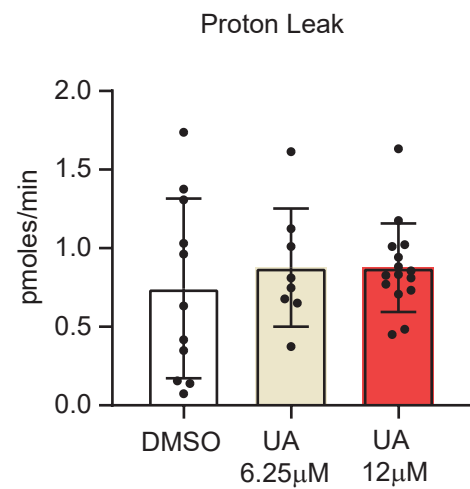**B**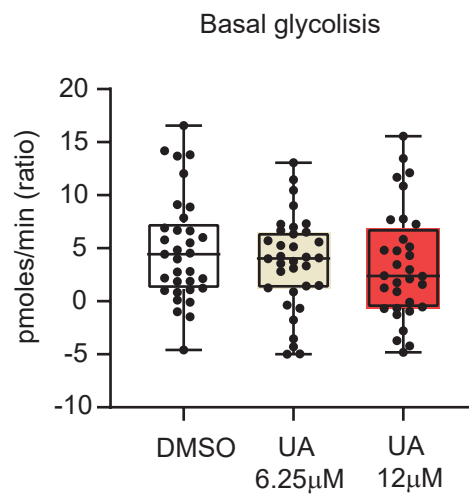**C**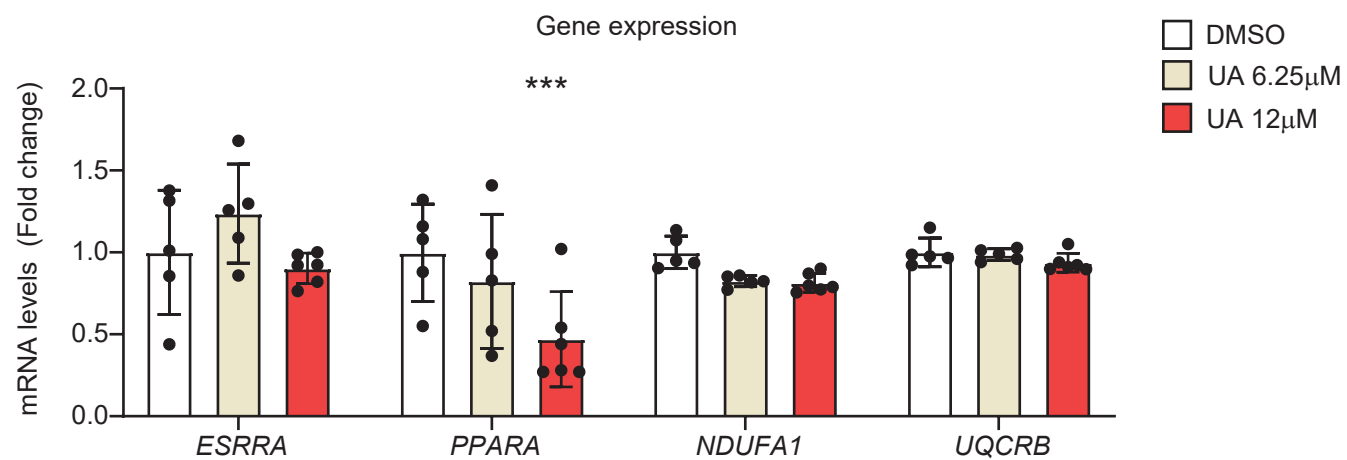

Supplement: Supplementary file 2 — Figure S1 [file ACEL-21-e13662-s003.pdf]

**A**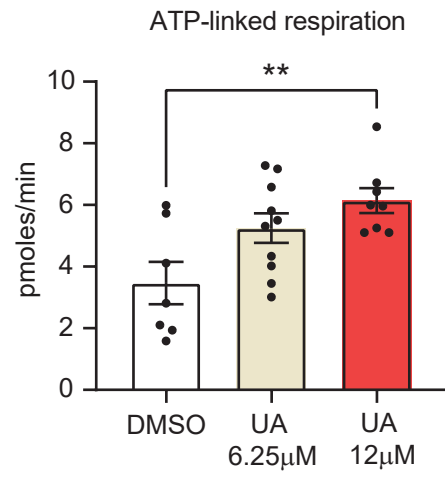**B**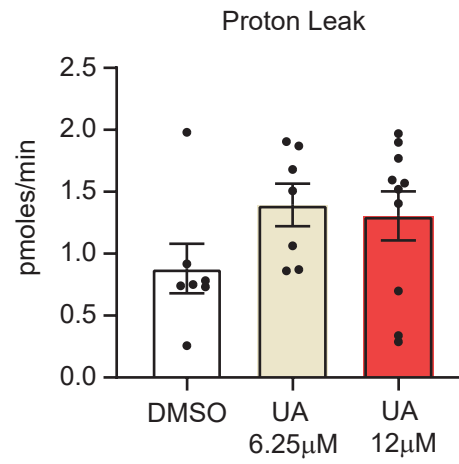**C**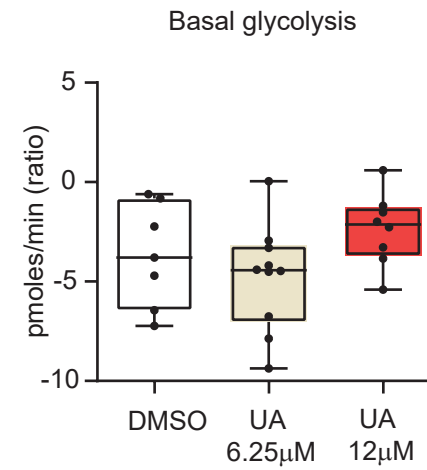

Supplement: Supplementary file 3 — Figure S2 [file ACEL-21-e13662-s001.pdf]

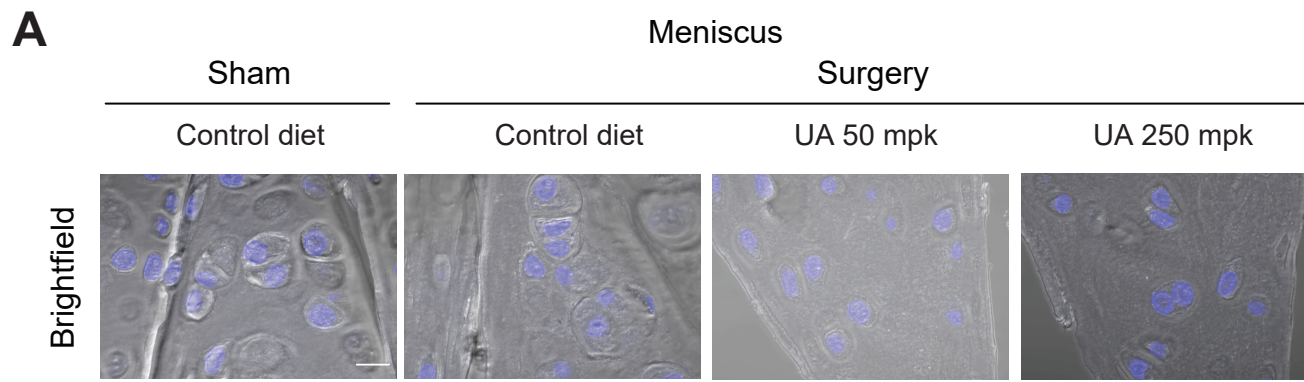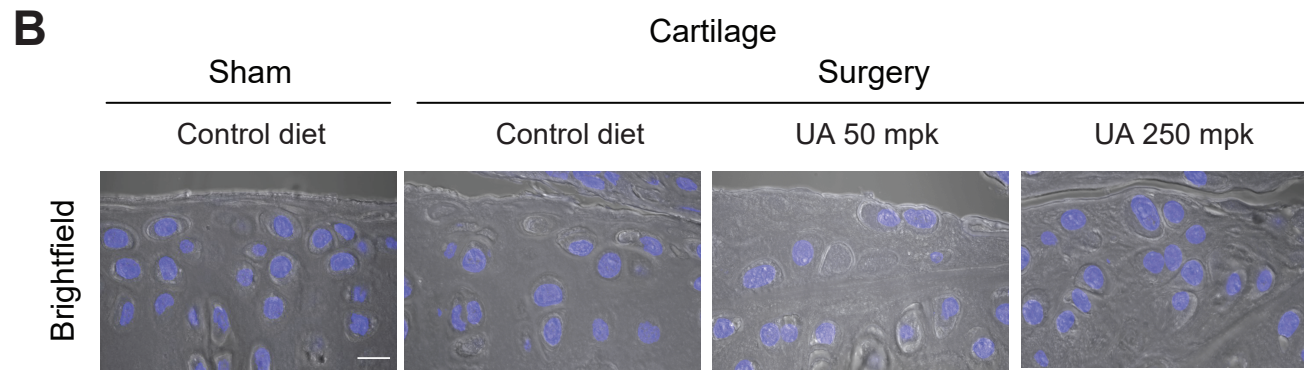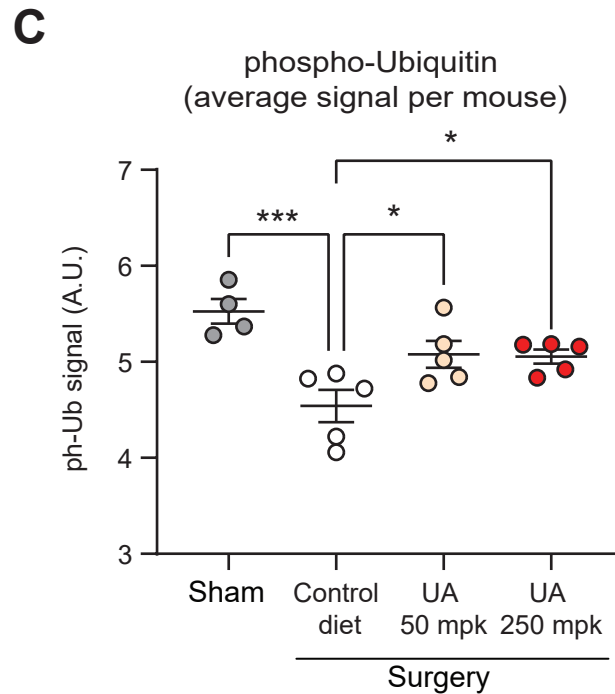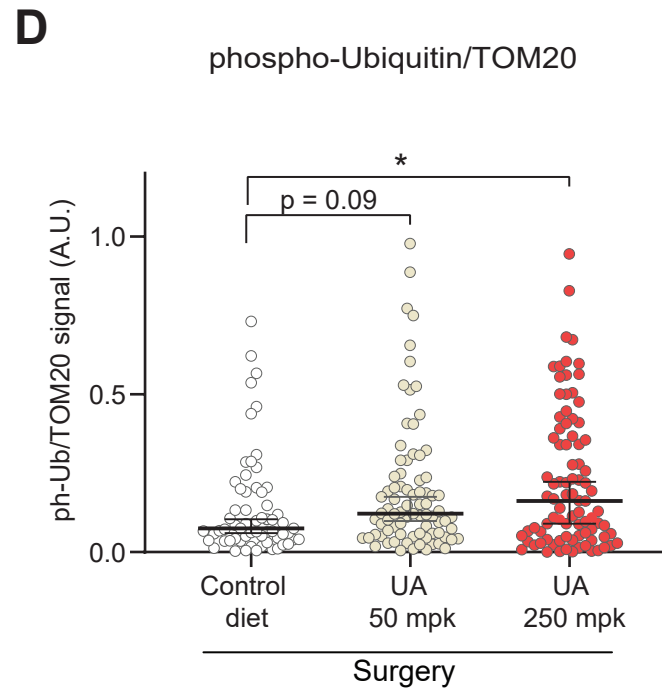

Supplementary Figure 3

Supplement: Supplementary file 4 — Figure S3 [file ACEL-21-e13662-s004.pdf]
